# How Chicago’s Past Resulted in Disproportionate Lead Poisoning of Minority Children of the Present: A Narrative Review

**DOI:** 10.7759/cureus.56694

**Published:** 2024-03-22

**Authors:** Glenn Goodwin, Todd Belok, Moshe Bengio, Bret Winners, Kevin Fan, Mitch Garey, Alexander J Scumpia, Erin M Marra, Laura Tortora

**Affiliations:** 1 Emergency Medicine, Aventura Hospital and Medical Center, Miami, USA; 2 Emergency, University of Illinois at Chicago, Chicago, USA; 3 Medical School, Nova Southeastern University Dr. Kiran C. Patel College of Osteopathic Medicine, Davie, USA; 4 Emergency Medical Services, Hatzalah South Florida, Miami, USA; 5 Emergency Medicine, Lakeside Medical Center - Health Care District Palm Beach County, Belle Glade, USA

**Keywords:** lead service lines, disproportionate lead poisoning, chicago lead poisoning, pediatric lead poisoning, lead poisoning

## Abstract

Chicago's lead problem has been shown to disproportionately affect populations of color and lower socioeconomic status (SES). The disproportionate effects on low-income areas and communities of color can be traced back to several key decisions in Chicago's history. A search of the National Library of Medicine’s MEDLINE/PubMed as well as Google, and Google Scholar was performed to find all articles relating to lead poisoning in Chicago, lead utilization, Chicago's municipal and political history, and lead physiology between May 2020 and May 2023. Additionally, several studies and textbooks were reviewed regarding the latest advancements in lead poisoning.

The study identified several key political moves over the course of Chicago's history that have resulted in disproportionate toxicity in minority populations and those of lower SES. Lead is more readily absorbed in the pediatric population. Additionally, prior regulations had published acceptable blood lead levels (BLLs) in children, but more recent evidence indicates a myriad of detrimental effects in BLLs below that cutoff. There is substantial evidence to suggest that there is no acceptable BLL. Lead toxicity is generally improving nationally but there still exists a considerable need for improvement. Programs should be expanded to ensure that individuals living in communities most at risk of lead exposure have the means to both, replace lead-contaminated infrastructure, and to be able to supply these communities with affordable housing. From a physician and clinician standpoint, knowing the increased risk of lead poisoning in these populations should prompt earlier testing.

## Introduction and background

Lead has long been a popular metal to use in the fabrication of pipes, as it is malleable, waterproof, and durable [1]. For this reason, over 70% of the largest cities in the United States had adopted lead-lined city-wide plumbing systems by the early 20th century, despite reports of lead’s potential toxicity since the Greco-Roman times [1]. As lead-lined service pipes became increasingly ubiquitous, reports of the toxicity of the drinking water grew in number and severity. Ultimately, by the 1930s the effects of lead toxicity had become scientifically accepted, leading to many states banning its use in service lines [1]. These restrictions, however, were a threat to the livelihoods of plumbers, whose expertise was installing lead-based pipes. In the late 1950s, Chicago Mayor Richard Daley saw strong plumber union support as key to his political success. With the help of Stephen Bailey, manager of the Chicago Plumber’s Union, a municipal code was passed requiring the use of lead for service pipes in the city [1]. Perhaps most importantly, it allowed only “licensed and bonded plumbers” to perform the installation of these pipes [1]. The Plumber’s Union ensured work and a monopoly on the maintenance of these pipes for its members. In return, Mayor Daley had shored up a sizable voting bloc that remained fiercely loyal [1]. The use of lead quickly proliferated in the city, leading to future ramifications for decades. Lead plumbing remained standard until the Safe Drinking Water Act was amended in 1986 [2]. That year, Chicago mayor Harold Washington outlawed the use of lead pipes and replaced them with plastic counterparts [2]. Despite the change, almost 400,000 lead service lines (LSLs) remain in use [3]. In 2016, the Illinois Environmental Protection Agency found Chicago had over twice the number of LSLs than any other city in America [4]. As infrastructure ages, these pipes have begun to leach lead into the water supply at alarming rates [5].

While the incidence of lead poisoning in the United States has drastically declined over the last several decades, most recent cases are disproportionally identified in lower-income communities, especially in the Chicago, Illinois area of the United States [6,7]. Like many environmental and medical problems facing society today, there is a disproportionate effect on lower socioeconomic status (SES) populations [6,7]. The authors examined the complex causes of this persistent concern, while also considering the intersection of racial, socioeconomic, and industrial forces that embody Chicago’s history. The focus of this paper was on the lead poisoning attributed to lead service lines (LSLs), as opposed to the more common causes such as dust and paint chips.

## Review

Methods

A search of the National Library of Medicine’s MEDLINE/PubMed, as well as Google Scholar, was performed with the goal of finding all articles published in the English language, utilizing some of the following keywords: “Chicago Lead poisoning,” “Lead service lines,” “Chicago Political history,” “Lead poisoning,” and “Lead Utility.” Additionally, a brief review of the latest articles on lead poisoning was performed and summarized. We mainly selected recent publications but did not exclude older works that were widely referenced. All data were accessed between May 2020 and May 2023. Our comprehensive PubMed/Medline search revealed a total of over 100 published manuscripts that mentioned an aspect of the parameters but not all were utilized in the finalized version of this review. The primary objective was to analyze the key factors in Chicago's history that have resulted in the contemporary lead problem, while also additionally providing an up-to-date review of lead pathology and guidelines, as there have been slight changes over the last several years. Given the nature of this manuscript, reputable news articles and substantiated press releases had to be utilized. We focused our search and data collection on lead poisoning secondary to service lines, rather than including lead exposure from dust and paint.

The study identified several key political moves over the course of Chicago's history that have resulted in disproportionate toxicity in minority populations and those of lower SES. We found that through key physiologic processes, lead is more readily absorbed in the pediatric population. Additionally, prior regulations had published acceptable blood lead levels (BLLs) in children, but more recent evidence indicates a myriad of detrimental effects in BLLs below that cutoff. There is substantial evidence to suggest that there is no acceptable BLL [6].

Mechanism of lead toxicity

While a full review of the toxicity of lead is out of the scope of this discussion, it is imperative to understand the mechanisms and clinical effects of lead poisoning to appreciate the significance of this issue on a public health scale. Features of plumbism are affected by multiple factors including the chemical composition of the toxic compound, the mode of absorption or exposure, the degree of exposure, the age of the exposed population, and chronicity of exposure, among others [6,8]. Lead is primarily absorbed via gastrointestinal and pulmonary systems [6]. Once absorbed, lead is distributed throughout the blood and soft tissues and is primarily stored in the bones, where it can remain for years [8]. While 95% of lead is stored in bone in adults, only about 70% of it can be found in the bones of children, leading to a relatively higher soft tissue burden in the pediatric population [8]. Consequentially, lead is more readily absorbed in children, leading to greater toxicity [8,9].

The biochemical properties of lead are responsible for its wide-ranging effects. It works at the sub-cellular level by interfering with processes that involve divalent cations, particularly calcium [10]. Lead’s effects on mitochondria have been linked to chronic nephrotoxicity [11]. Lead’s direct genotoxic effects, inhibition of ferrochelatase (a key enzyme in heme synthesis), as well as decreased erythropoietin release contribute to hematologic toxicity and lead-induced anemia [12]. Direct inhibition of the enzyme pyrimidine-5’-nucleotidase causes the basophilic stippling seen by microscopy in red blood cells [13]. Lead’s ability to modulate calcium-dependent processes, damage endothelium, and alter endocrine systems may lead to potential direct cardiac injury, thrombogenesis, ischemic heart disease, and chronic hypertension [14]. In the skeletal system, alterations in bone formation lead to pathologic fractures and osteoporosis [6]. Furthermore, it is considered by the International Agency for Research on Cancer to be a 2A carcinogen, indicating that it is probably carcinogenic to humans [15,16]. The nervous system is among the hardest hit by lead toxicity. Massive lead exposures with markedly elevated BLLs, rare in the United States, may lead to potentially life-threatening encephalopathy, seizures, coma, and ultimately death and require intensive monitoring and emergent chelation [6,8,12,13].

When discussing lead toxicity on a public health scale, particular attention should be paid to the delayed, chronic effects of low-level lead exposure in childhood. Lead exposure has been long associated with negative cognitive and developmental effects even when the exposure is relatively minute and when symptoms are subtle [17]. Malnourishment, as well as deficiencies in calcium, iron, and zinc, can lead to increased GI absorption of lead, likely secondary to decreased competitive absorption of these elements by the divalent metal transporter in the duodenum [17]. These effects are likely to be even more apparent in a low-income population struggling with food insecurity [18]. While past studies have focused on identifying a so-called “blood lead level of concern,” data instead has indicated that there is no safe BLL in children, a position supported by the Advisory Committee on Childhood Lead Poisoning Prevention [19]. Alarmingly, studies have demonstrated, an average IQ decrease of about 2-4, for every microgram per deciliter BLL elevation [17]. Even BLLs under 7.5 μg/dL result in major IQ reductions [20]. Equally alarming concern for the development of attention deficits and behavioral issues even in children whose levels are considered “safe.” A resolution of potential lead exposures in children is prudent from an ethical, public health perspective [21].

Impact on low-income communities

The disproportionate effects on low-income areas and communities of color can be traced back to several key decisions in Chicago's history. In the late 1990s and early 2000s, Chicago Mayor, Richard M. Daley, worked to transition public housing away from large high rises and into private homes, based on the theory that large public housing complexes facilitated criminal gang activity [22]. Housing projects were demolished, and the Housing Choice Voucher (HCV) program was started [9]. The HCV program provides stipends in the form of vouchers, which can then be used by tenants to rent private properties [9]. Though well-intentioned, this program led to the creation of de facto housing projects where landlords rent out entire buildings, essentially replacing the large public housing complexes with large private ones [23]. Private landlords receiving these modest vouchers are discouraged from major and cost-prohibitive renovations, thereby keeping the preexisting LSLs in place. Nearly 95% of the remaining LSLs are located in low-income communities [23].

BLL cutoffs

The Chicago Housing Authority is required to inspect rent-subsidized homes annually, assessing for potential lead exposure sources and various other hazards [10]. These inspections are only required to report obvious defects such as chipped paint and do not assess lead levels in dust or water [10]. The federal government has made efforts to combat this issue by mandating landlords to replace lead lines based on the BLL of the inhabitants, but initially set a serum lead cutoff that was excessively high [1,2]. Landlords were not required to make any renovations or changes until tenants had evidence of a BLL as high as 20 µg/dL. Eventually, this cutoff was reduced to 5 µg/dL [24].

While this improvement is substantial, it still cannot reverse the damage done by the initially high cutoff. The lead burden borne by impoverished communities of color is not limited to the city of Chicago. A report by the Center for Disease Control reviewed data from the National Health and Nutrition Examination Surveys (NHANES) between 1999 and 2002 regarding the incidence of patients with BLLs >10 µg/dL [1]. Data showed that elevated BLLs were significantly more likely in people identifying as Black non-Hispanic than in other populations [1]. A separate CDC report found that children enrolled in Medicaid had a prevalence of elevated blood lead concentrations three times greater than those not enrolled [2].

Lead service replacement program

In response to these alarming statistics, Chicago Mayor Lori Lightfoot announced the Lead Service Replacement Program (LSLRP) in 2016 [4]. With an estimated cost of $8.5 billion, this program is the most significant attempt by the city to address the LSL issue [4]. The LSLRP takes a three-pronged approach by which the city will provide LSL replacement for low-income homeowners, waive permit fees for residents who pay to replace their own service line, and replace the full water main in select city blocks [4]. While the good intentions of this program cannot be understated, it unfortunately has a major drawback: residents must own and live in the LSL homes, which excludes anyone in the HCV program. Under this program, a landlord may not be eligible for assistance in removing LSLs even if low-income residents are being exposed to chronically high levels of lead.

Limitations

The nature of this review lends itself to several limitations, particularly pertaining to bias. The nature of political history makes complete eradication of bias difficult. Sources and claims were corroborated but some of the lesser-known reasons behind historical accounts can be difficult to definitively prove. Many of the accounts were obtained through old news sources which often do not adhere to the same rigorous standards as a peer-reviewed journal. We also focused our assessment on lead toxicity secondary to LSLs. The more common causes of lead toxicity such as chipped paint and dust certainly play a role in confounding some of our findings in rates of toxicity.

## Conclusions

Lead has serious health and cognitive effects and disproportionately affects low socioeconomic communities. Much work has been done to eliminate lead from the community, such as legislation requiring LSL renovations. Initiatives such as the Lead Service Replacement Program are a good first step towards providing incentives to replace LSLs but it must be expanded to encourage LSL replacement in landlord-owned properties. The first step to solving the lead crisis must therefore be to acknowledge that those most affected by plumbism are the same communities marginalized by centuries of social and economic inequity. Programs should be expanded to ensure that individuals living in communities most at risk of lead exposure have the means to both replace lead-contaminated infrastructure and to be able to supply these communities with affordable housing without risk of toxic exposure. From a physician and clinician standpoint, knowing the increased risk of lead poisoning in these populations should prompt earlier testing in the case of the aforementioned signs and symptoms. There is no acceptable BLL, so any lead discovered in the serum warrants an environmental investigation. Earlier recognition and diagnosis of lead poisoning, perhaps by routine screening exams, will lead to greatly improved patient and societal outcomes.
